# The Use of Clay-Polymer Nanocomposites in Wastewater Pretreatment

**DOI:** 10.1100/2012/498503

**Published:** 2012-02-15

**Authors:** Giora Rytwo

**Affiliations:** ^1^Environmental Physical Chemistry Laboratory, MIGAL, Galilee Technology Center, Kiryat Shmona 11016, Israel; ^2^Department of Environmental Sciences, Tel Hai College, Upper Galilee 12210, Israel

## Abstract

Some agricultural effluents are unsuitable for discharge into standard sewage-treatment plants: their pretreatment is necessary to avoid clogging of the filtering devices by colloidal matter. The colloidal stability of the effluents is mainly due to mutual repulsive forces that keep charged particles in suspension. Pretreatment processes are based on two separate stages: (a) neutralization of the charges (“coagulation”) and (b) bridging between several small particles to form larger aggregates that sink, leaving clarified effluent (“flocculation”). The consequent destabilization of the colloidal suspension lowers total suspended solids (TSSs), turbidity, and other environmental quality parameters, making the treatments that follow more efficient. Clay-based materials have been widely used for effluent pretreatment and pollutant removal. This study presents the use of nanocomposites, comprised of an anchoring particle and a polymer, as “coagoflocculants” for the efficient and rapid reduction of TSS and turbidity in wastewater with a high organic load. The use of such particles combines the advantages of coagulant and flocculant by neutralizing the charge of the suspended particles while bridging between them and anchoring them to a denser particle (the clay mineral), enhancing their precipitation. Very rapid and efficient pretreatment is achieved in one single treatment step.

## 1. Introduction

Water from some agricultural effluents (such as olive mills, wineries, piggeries, soy, or coffee bean industry) is unsuitable for discharge into standard sewage-treatment plants, due to the large amounts of organic and suspended matter. The annual production of black and highly toxic olive mill wastewater in the Mediterranean olive-growing countries is estimated at about 10–30 million m^3^. The disposal of such effluents without any treatment is known to cause serious environmental problems [1]. For example, in Israel, in the winter of 2006-2007, olive mill wastewater effluents seriously impacted the water quality of several springs along the Keziv stream (Elhanani, personal communication); many other instances have gone unreported [2]. Wineries are also major producers of organically laden wastewater, yielding about 1000–3000 L per ton of grapes [3], characterized by high contents of organic material and nutrients, high acidity, and large variations in seasonal flow production [4]. The very high values of organic matter, suspended solids, and sodium adsorption ratio (SAR) make such water inadequate for disposal in common sewage systems [5].

Colloidal particles that tend to clog filtering devices are one of the problems with such effluents [6]. In most cases, colloidal stability (i.e., the colloids' tendency to remain dispersed) of the effluents is due to three factors.

- Very small particles do not tend to sink. For a given type of particle in a given liquid, sedimentation rate is proportional to the particle's diameter squared [7]. This is because the particle's mass (and thus its weight, pulling it “down” and making it sink) depends on its diameter cubed. On the other hand, the frictional force working against the particle's movement (Stoke's Law) increases with actual diameter [8]. Thus for small particles, the weight is too small to overcome the drag force, yielding very low sedimentation rates.
- The weight of the particle also depends on its density. The density difference between the particle and the fluid affects the sedimentation rate [9]. In organic effluents most suspended solids are organic matter, with a density close to that of water, avoiding their separation from the liquid.
- In most cases colloidal particles are electrostatically charged. In mineral colloids those charges occur due to imperfections in the lattice of the minerals, whereas on organic matter such charges might derive from functional charged groups (amines, carboxyl, phosphate, etc.) [8]. Electrostatic repulsion between the particles avoids its aggregations, stabilizing the colloidal dispersion.

Although the terms “coagulation” and “flocculation” are sometimes used interchangeably, they can be ascribed to two different stages. Coagulation indicates the process through which colloidal particles and very fine solid suspensions are *destabilized *so that they can begin to agglomerate if conditions are suitable. Flocculation refers to the process by which destabilized particles actually *conglomerate *into larger aggregates so that they can be separated from the wastewater [10]. To achieve these effects, a series of industrial steps are used. In most cases, the treatments are based on two separate stages, performed in two separate tanks: (a) neutralization of the charges to overcome electrostatic repulsion (yielding “coagulation”) and (b) bridging between several relatively small particles to form larger aggregates that, due to their size and density, sink to the bottom of the vessel, leaving a clarified effluent (leading to “flocculation”). Addition of “coagulant” and “flocculant” together usually yields an ineffective process due to binding between the additives. Thus, destabilization of the colloidal suspension, followed by flocculation of large amounts of suspended matter, lowers the values of total suspended solids (TSSs), turbidity, and even chemical oxygen demand (COD). This, in turn, improves the efficiency of following water treatments, thereby reducing environmental hazard.

In most cases, pretreatment processes involve the use of chemicals for neutralization, flocculation, and precipitation of colloids in wastewater. Anastasiou et al. [11] prepared a complex system for winery effluents that included passing them through a screen, a bioreactor, coagulation, and flocculation. The result was an 85% reduction in TSS in 20 d. Similar results were obtained using chitosan (a biopolymer) as the coagulant, for both olive mill and winery effluents [12]. Physical separation has also been proposed: studies based on nanofiltration [13] or reverse osmosis [14] have reported 95% TSS removal. However, the procedure is expensive, and the resultant concentrated effluents (comprising 10–20% of the volume, with very high TSS) still need to be treated. 

Clays and organoclays (clay minerals treated with organocations) have been widely used for the pretreatment of effluents [15, 16]. Several studies have used cationic or anionic polyelectrolytes [17], combinations of coagulants and polyelectrolytes [18], or even combinations of clay minerals and organic quaternary ammonium ions [19] for the removal of organic contaminants from olive mill wastewater. In all cases, considerable changes in the colloidal properties of the effluent, including reduction in turbidity, TSS, COD, and other parameters were achieved. 

The term “nanoparticle” is usually used for a combined material which has at least on one dimension a size of 100 nm or less [20]. Thus, most clay minerals are considered nanoparticles. Nanocomposite materials consisting of polymer molecules and natural or layered minerals like clays can be prepared by adjusting the interaction enthalpy between all components [21]. The use of clays as building blocks for assembling organic species at the nanometer range yields useful hybrid nanostructured materials. Such nanocomposites can be prepared and designed by the combination of clay minerals with organic species interacting at the molecular level [22].

In previous studies, we demonstrated the ability of suitable nanoparticles for very efficient removal of phenolic compounds similar to components of olive mill or winery wastewater [23]. Other studies [6, 24] presented a very effective pretreatment based on combinations of organoclay nanoparticles and crude clay, which changed the colloidal stability of winery and pickle industry effluents, reducing TSS and turbidity for several cycles by means of a two-step process: a first step performed with an organoclay and a second step performed by adding raw clay. In general, that process was similar to those used today in the industry: (a) a coagulation step, performed in the industry with cationic polymers, or with aluminum sulfate or other inorganic polycations (in the above report, the coagulant was based on an organoclay) and (b) a flocculation step performed in the industry with flocculants which are, in some cases, based on cationic or anionic polyacrylamide derivatives (in the above report, the flocculant was a raw clay mineral). The improvement proposed by Rytwo et al. [24] lays in the possibility of performing several cycles with the same amount of additives.

In the present study, suitable nanocomposites (based on low-cost clay minerals and organic molecules or polyelectrolytes) adapted to the charge of the effluent are shown to provide a very efficient pretreatment for the reduction of TSS and COD in a single step. Nanocomposites presented in this study are based on the clay mineral sepiolite and the cationic polymer poly-DADMAC. However, the general idea (as presented in [25]) behind the use of nanocomposites is to combine neutralization of the colloids (coagulation), achieved by the polymer's charged sites, and bridging of the neutralized particles (flocculation), achieved by the fact that the polymer chains are connected to denser and heavier anchoring particles; this might yield very efficient pretreatment of wastewater and the reduction of TSS in one single treatment step.

Thus, since stability of a dispersion is related to the size of the its particles, their density, and their charge and considering that in organic effluents the colloids usually have a negative charge, a cationic polymer should be used to neutralize the charge of the colloids. In addition, the polymer should have medium to long chains with charges dispersed throughout to allow the bridging effect. The polymer should also be relatively soluble in water to allow its efficient distribution in the effluents. Accordingly, suitable polymers might be (i) a linear water-soluble polymer such as poly-DADMAC or cationic polyacrylamide; (ii) polyquaternium molecules such as quaternized hydroxyethylcellulose ethoxylate; (iii) cationic biopolymers such as cationic guar gum or chitosan; (vi) polymers with aromatic rings such as poly-4-vinylpyridine-co-styrene and additional styrene-based cationic copolymers. The latter might even yield additional *π*-*π* interactions with organic colloids, allowing for more efficient coagoflocculation.

For efficient coagoflocculation the anchoring particles should have the following properties: (i) a size/diameter of less than 0.5 *μ*m in at least one dimension, resulting in a large specific surface area; (ii) the ability to adsorb cationic polymers in strong interactions; (iii) the bulk density of the particles should be larger than the density of the effluents. Accordingly, anchoring particles might be acicular (needle-like) clay minerals such as sepiolite and palygorskite or clay smectites such as montmorillonite, hectorite, laponite, and saponite. However, similar results might be obtained with nonclay minerals such as zeolites or even powdered activated carbon.

**Figure 1:**
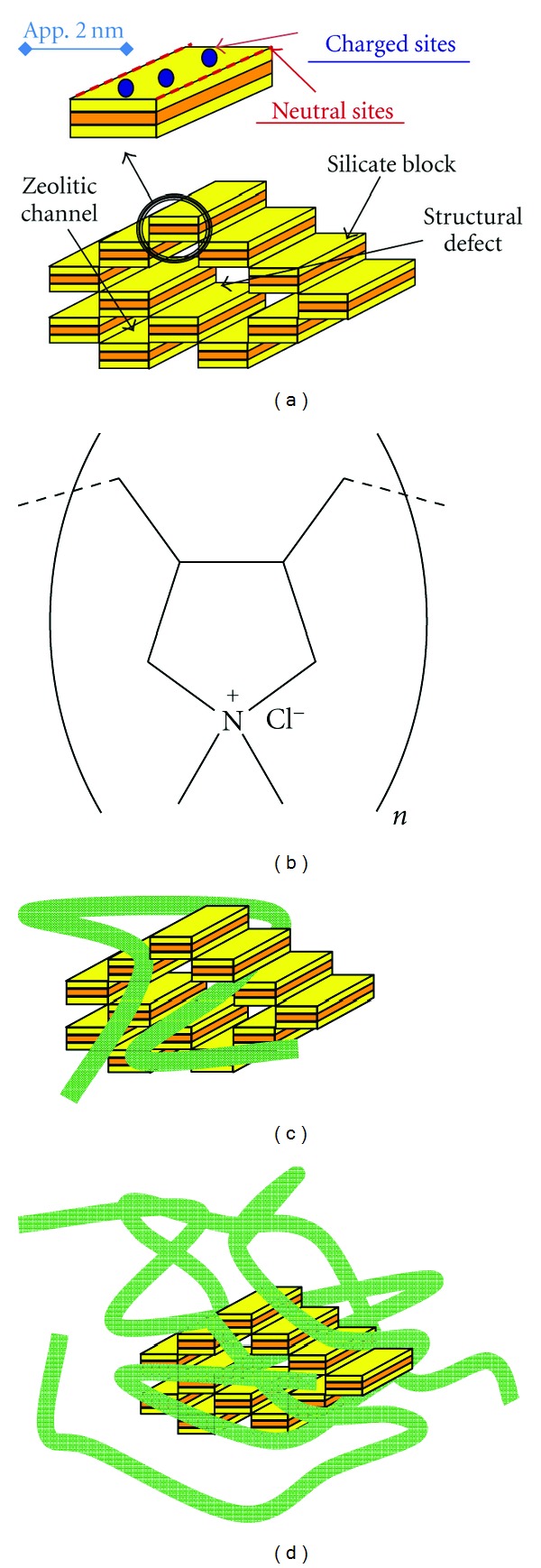
Schematic structure of (a) a single block and connected blocks of sepiolite. The 2 nm size bar is given as a relative dimension; (b) poly-DADMAC monomer molecule; (c) low-charge nanocomposite suitable for winery effluents. The ribbons illustrate the polymer chains with positive charges distributed throughout; (d) high-charge nanocomposite suitable for olive mill effluents.

In this study, the nanocomposites were based on the needle-like clay mineral sepiolite as the anchoring particle and the linear cationic polymer poly-DADMAC as the bridging ribbon. The general structure of sepiolite consists of alternating blocks and tunnels that grow in the direction of the fiber (Figure 1(a)). All corners are connected to adjacent blocks, but in outer blocks some of the corners form neutral sites accessible to organic molecules. In addition to that, some isomorphic substitutions in the lattice of the mineral form negatively charged adsorption sites. These characteristics of sepiolite make it a powerful sorbent [26]. Poly-DADMAC is a homopolymer used in effluent treatment, water purification, and paper industry (Figure 1(b)).

Since the proposed nanocomposites act as coagulants by neutralizing charged colloidal particles and as flocculants by bridging the neutralized particles together to form larger and denser aggregates that sink relatively rapidly, the term “coago-flocculant” was proposed [25]. Adsorption of phenolic compounds by similar nanocomposites has been demonstrated [27], with very fast sorption kinetics, similar to values shown previously for organoclays, and two or three orders of magnitude faster than activated carbon [27, 28]. However, removal of phenolic compounds is only one beneficial side effect. The main purpose of the coago-flocculant presented in this study is to achieve a two-order of-magnitude reduction in TSS and turbidity in a very short time (minutes to tens of minutes) in a single application. The nanocomposites used need to be adapted to specific effluents, but the choice of a suitable nanocomposite is easily made by preliminary calibration experiments using suitable instruments as presented below. In the lack of such instrument, similar calibration experiments can be performed using conventional “jar test” procedures [29].

## 2. Materials and Methods

### 2.1. Materials

Olive mill wastewater was kindly supplied by Ein Kamonin Olive Mill (Lower Galilee, Israel). Winery effluents were obtained from Galil Mountain Winery (Yiron, Upper Galilee, Israel). Sepiolite S9 (<200 mesh) was provided by TOLSA S.A. (Madrid, Spain), with 99% pure mineral content, and polydiallyl dimethylammonium chloride (poly-DADMAC; medium molecular weight, 200.000 to 350.000) was purchased from Sigma-Aldrich (Israel). All materials were used without further treatment or purification.

### 2.2. Preparation of Nanocomposites

Nanocomposites were prepared from sepiolite and poly-DADMAC at loads ranging between 3 and 2400 mg polymer/g clay. Concentrated batches containing 100 g clay/kg (10%) suspension were prepared. To produce the nanocomposites, a solution containing the requested amount of polymer was prepared according to the desired amount of polymer per gram of clay. As an example, the procedure for preparing a 10% stock suspension of 50 g nanocomposite with 100 mg poly-DADMAC/g sepiolite was as follows. The concentrated polymer (poly-DADMAC, usually 40% w/w) was dissolved in a suitable amount of warm water to obtain a final volume of 500 mL containing 5 g of the polymer. The solution was placed in a sonication bath to obtain a homogeneous solution. Upon complete dissolution, the polymer solution was poured into a container with 50 g of sepiolite and agitated vigorously for 2 h. Preparation was complete when clay aggregates were no longer observed, and the viscosity of the suspension was relatively low. Increased viscosity indicates that the polymer is not well dissolved or that the process is not yet complete, since a 10% suspension of most clay minerals in water (without polymer) yields a paste that cannot be efficiently used.

### 2.3. Analytical Measurements

#### 2.3.1. Electrokinetic Charge Measurements

Electrokinetic charge of the nanocomposite suspensions and the effluents before and after treatment were measured by means of a particle charge detector (Mütek PCD 03) with an automatic titration unit (Mütek titrator T2) using charge-compensating polyelectrolytes as described by Rytwo et al. [6]. Electrokinetic effects occur whenever there is a distortion of counter ions due to movements of charged particles relative to the surrounding solution, and are they are widely used to characterize the charge distribution around colloidal particles in an aqueous solution. Results were normalized to *μ*mol_c_/g (micromoles of charges per gram) of nanocomposite or to mmol_c_/L of effluent, accordingly. All experiments were performed in triplicate.

#### 2.3.2. Harmonic Mean Sedimentation Velocity

The presented nanocomposites were tested for their influence on the sedimentation rate of olive oil mill and winery wastewaters. Sedimentation velocities were measured by means of a LUMiSizer instrument. The instrument records the near-infrared light transmission during centrifugation over the total length of a cell containing the suspension. It automatically determines the time dependence position of the interface panicle-free fluid/suspension or sediment by a special algorithm. The transmission profile enables characterizing the smallest deviations in size of dispersed particles and quantifying the degree of polydispersity at high-volume concentrations. Stability prediction at an accelerated rate for different dispersions at their original concentrations has been proven in previous studies [9]. The harmonic mean sedimentation velocity in the first 60 seconds of the process was chosen as a useful parameter to compare between treatments. High sedimentation velocities were measured when fast precipitation was observed. The reason to focus on the first 60 s is because in the efficient treatments, complete clarification was observed after that period of time. Such experiments allow evaluating the efficiency of the wastewater treatment by a very fast and accurate procedure as compared with the conventional “jar test” [29]. Experiments were performed three times.

#### 2.3.3. Flocculation Kinetics in Winery Effluents

To compare the efficiency of flocculation of nanocomposites with other widely used treatments, 0.1% nanocomposite with 60 mg poly-DADMAC/g sepiolite was added to winery effluents, and the relative absorbance of the effluent (the ratio of the optical density at a given time related to the optical density before the addition of coagulant/flocculant) was compared to that of the nanocomposite components (sepiolite and poly-DADMAC) separately at the same added amounts of active compound. The efficiency of flocculation by 0.1% aluminum sulfate (alum) was also determined for comparison, since this is a widely used flocculant. Measurements were performed at three wavelengths (420, 450, and 480 nm), and the average relative absorbance values were evaluated.

#### 2.3.4. Batch Precipitation Reactors

After determination of the optimal polymer/clay ratio for each effluent, larger volume vessels were prepared. For this purpose, 250-mL laboratory vessels were filled with 200 mL effluent and the corresponding amount (1 mL) of 10% nanocomposite suspension was added to a final ratio of 0.05% clay. The vessel was stirred with a magnetic stirrer at maximum speed for about 1 min, and then for an additional 2 min at low speed. Flocs were left to precipitate for 30 min. Turbidity was measured with a LaMotte 2020i turbidimeter, and electrical conductivity and pH were measured with an EXTECH EC500 pH/conductivity probe. TSSs were measured according to standard method 2540 [30], and electrokinetic charge was measured as described above. All parameters were measured before and after treatment.

## 3. Results and Discussion

**Figure 2:**
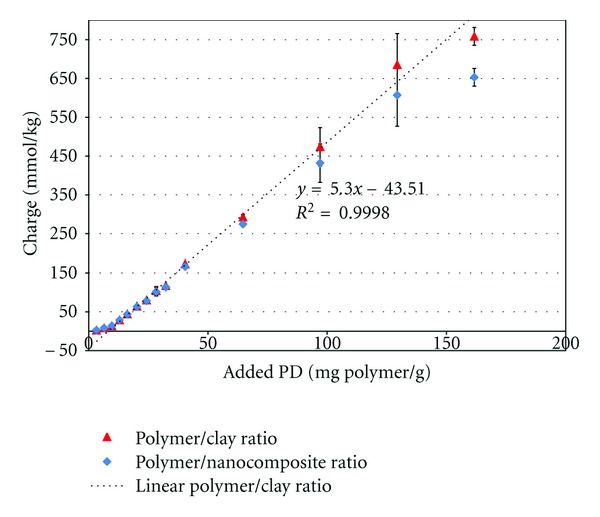
Charge of the nanocomposites as a function of polymer/clay ratio (triangle) and polymer/nanocomposite ratio (rhombus).

The charge of the nanocomposite suspensions measured by particle charge detector is shown in Figure 2. Results are presented as a function of the polymer/clay ratio and the polymer/total nanocomposite weight ratio. A linear correlation can be seen between the charge of the particles and the polymer/clay ratio. The same correlation occurred with larger amounts of polymer in the composite, with a slope of 5.3 mmol_c_/g polymer. When transforming this value per mole of monomer, the slope was 0.854 mol_c_/mol polymer, indicating that approximately 85% of the monomer units in the bound polymer are charged. The correlation between the charge and the polymer/nanocomposite ratio was not linear because at high amounts of added polymer, a considerable part of the weight of the nanocomposites comes from the polymer itself.

**Figure 3:**
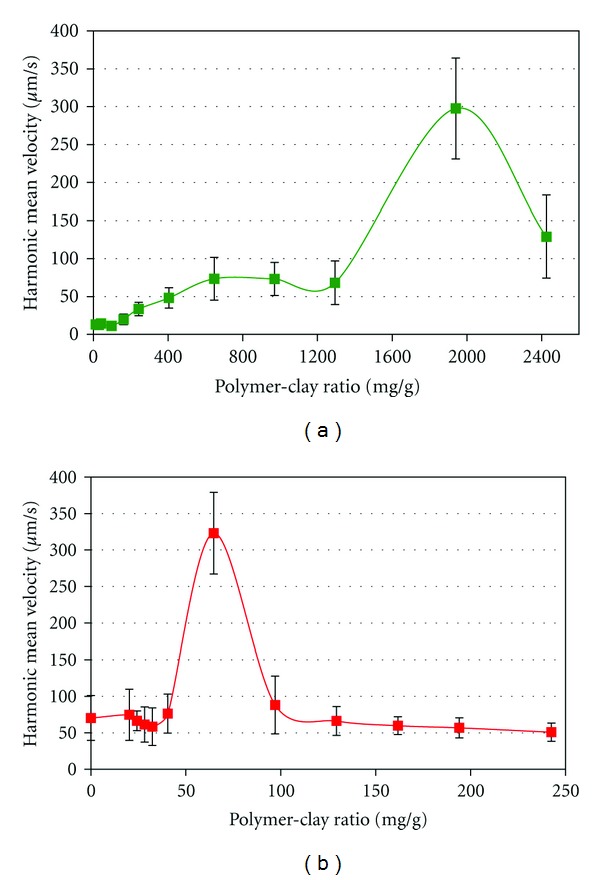
Harmonic mean sedimentation velocity of effluents upon addition of 0.1% poly-DADMAC-sepiolite nanocomposite as a function of the polymer/clay ratio. (a) Olive oil wastewater; (b) winery wastewater.

The nanocomposites were tested for their effect on sedimentation rate of olive oil mill and winery wastewaters (Figure 3). The winery wastewater sedimentation rate was increased almost 10-fold by adding nanocomposites with approximately 70 mg polymer/g clay. Olive mill wastewater is considerably more charged (see Table 1), and nanocomposites that increased sedimentation rates in winery wastewater were not efficient in this case. However, highly charged nanocomposites (Figure 1(d)) with 1800 mg polymer/g clay sped up sedimentation to more than 10-fold the rate of raw effluents.

Table 1 shows the changes in the physicochemical parameters in batch experiments, before and 30 min after addition of the suitable nanocomposites to each effluent. Whereas the pH remained unchanged and the electrical conductivity was reduced by 5–20%, complete neutralization and even charge reversal of both effluents (up to values of about 5% of the absolute initial charge) were observed. The more important feature of the nanocomposites was a 97% reduction in TSS and turbidity in less than 1 h. It should be mentioned that a 90% reduction in total Kjeldahl N and a 40% reduction in COD was also measured (Shifron, Rytwo and Litaor, 2011, unpublished results).

**Figure 4:**
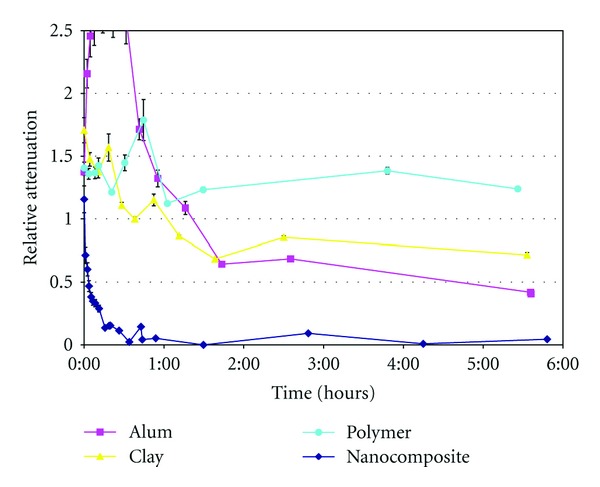
Relative light attenuation of winery effluents as a function of time, upon addition of 0.1% alum, 0.1% poly-DADMAC/sepiolite nanocomposite, and the equivalent amounts of sepiolite (clay) or poly-DADMAC (polymer) separately.

Figure 4 shows the flocculation efficiency of 0.1% nanocomposite with 60 mg poly-DADMAC/g sepiolite in comparison to that of its separate components (sepiolite and poly-DADMAC) at the same added amounts of active compound. The efficiency of flocculation of 0.1% alum was also determined for comparison. Strong temporal fluctuations were due to large flocs, which could be clearly seen with the naked eye, that initially floated up in the tube and eventually sank to the bottom. The nanocomposites achieved almost complete clarification in a range of minutes. It should be emphasized that at long equilibration times (24 h), clay and alum treatments produced more or less the same results, whereas the polymer alone at that added rate did not enhance clarification. However, the observed rapid (on the order of minutes) flocculation is the main feature of the nanocomposites: this might enable continuous flow in treatment plants, thereby eliminating the large sedimentation tanks necessitated by the long periods required for sedimentation to occur. 

**Figure 5:**
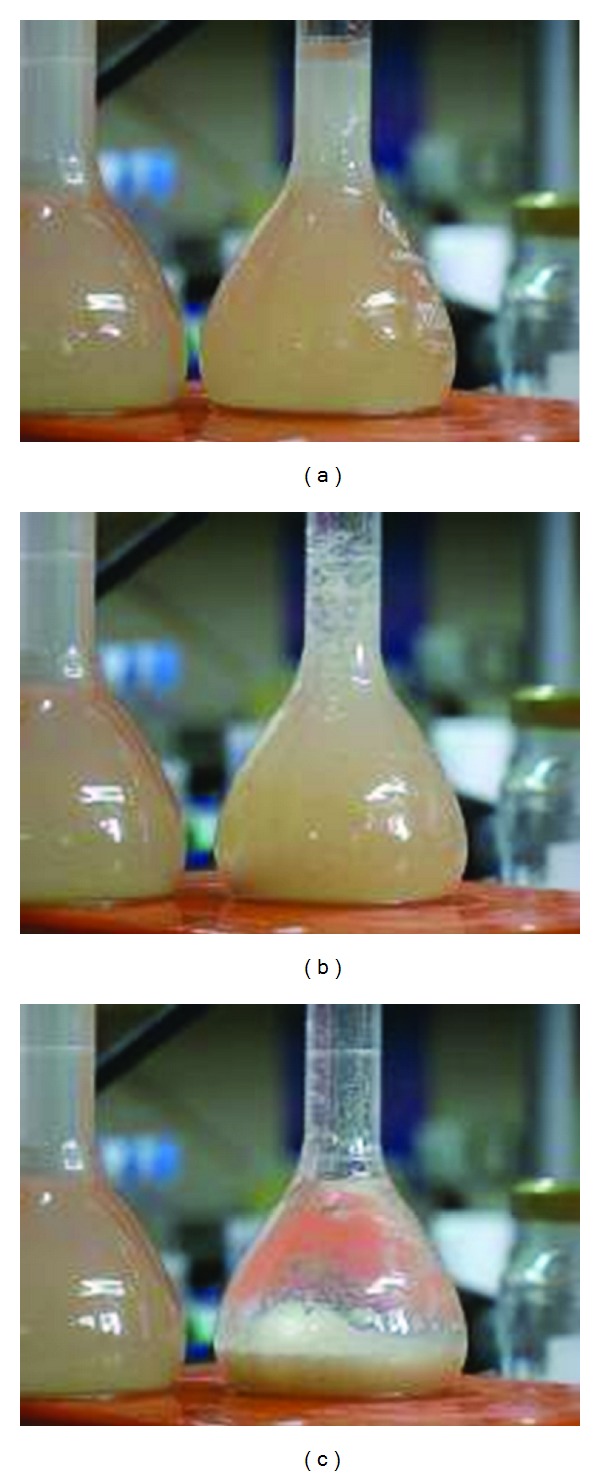
Winery effluents upon addition of 0.1% poly-DADMAC/sepiolite nanocomposite, (a) at the time of addition, (b) 45 s after addition, and (c) 90 s after addition. In all panels, the bottle on the left contains untreated effluents.

Figure 5 demonstrates the rapid sedimentation upon addition of suitable nanocomposites to winery effluent. In this case, 70 mg polymer/g clay nanocomposites were used. Complete clarification of the winery effluent was obtained in less than 2 min (a video clip is available at the Journal website).

## 4. Conclusions

This study demonstrates that very rapid sedimentation of effluents with a heavy organic load can be achieved by means of suitable clay-polymer nanocomposites. 

The main features of this treatment are as follows.

- It is performed in one step, combining flocculation and coagulation in one tank, in a matter of minutes.
- Raw materials are relatively inexpensive (ubiquitous clay minerals and widely used polymers) and environmentally friendly, since the use of biopolymers can be encouraged.
- Preparation is simple.
- The charge of the nanocomposites must be adapted to the effluent's charge. Unsuitable particles will not yield efficient flocculation. However, since measurement of effluent charge is a simple procedure, this is not expected to present a major problem.

It should be emphasized that variability in effluents (especially in olive mills) is enormous, with TSS values ranging between 1000–100000 mg/L, and in some cases up to 1 : 5 dilution might be needed. However, the results presented here show that the proposed nanocomposites can speed up the precipitation process. By reducing clarification time from several hours to minutes, the need for large precipitation tanks, as required for slow treatments, might be avoided, making the wastewater treatment an almost continuous process. Since the sludge obtained is more than 99% organic matter, additional studies are performed on composting processes that may yield material usable as soil conditioner.

## Figures and Tables

**Table 1:** Physicochemical parameters of effluents upon addition of 0.05% of a suitable coago-flocculant.

Effluent		pH	Electrokinetic charge *μ*mol_c_/L	Electrical conductivity *μ*Si/cm	Turbidity NTU	TSS mg/L
Winery	Untreated	4.66	−174	2460	764	1610
	Treated after 30 min	4.69	+10.1	2040	19.6	76
Olive mill	Untreated	4.40	−48400	8160	>4000	1880
	Treated after 30 min	4.38	+920	7680	91	94
